# Rab geranylgeranyl transferase β subunit is essential for male fertility and tip growth in *Arabidopsis*

**DOI:** 10.1093/jxb/eru412

**Published:** 2014-10-14

**Authors:** Malgorzata Gutkowska, Marta Wnuk, Julita Nowakowska, Malgorzata Lichocka, Michal M. Stronkowski, Ewa Swiezewska

**Affiliations:** ^1^Institute of Biochemistry and Biophysics, Polish Academy of Sciences, Pawińskiego 5a, 02-106 Warsaw, Poland; ^2^Warsaw University, Faculty of Biology, Miecznikowa 1, 02-096 Warsaw, Poland; ^3^Warsaw University of Technology, Faculty of Mathematics and Information Science, Koszykowa 75, 00-662 Warsaw, Poland

**Keywords:** *Arabidopsis*, male fertility, pollen, pollen tube, protein prenylation, Rab geranylgeranyl transferase, Rab protein, root hair, tip growth.

## Abstract

Rab proteins, key players in vesicular transport in all eukaryotic cells, are post-translationally modified by lipid moieties. Two geranylgeranyl groups are attached to the Rab protein by the heterodimeric enzyme Rab geranylgeranyl transferase (RGT) αβ. Partial impairment in this enzyme activity in *Arabidopsis*, by disruption of the *AtRGTB1* gene, is known to influence plant stature and disturb gravitropic and light responses. Here it is shown that mutations in each of the *RGTB* genes cause a tip growth defect, visible as root hair and pollen tube deformations. Moreover, FM 1–43 styryl dye endocytosis and recycling are affected in the mutant root hairs. Finally, it is demonstrated that the double mutant, with both *AtRGTB* genes disrupted, is non-viable due to absolute male sterility. Doubly mutated pollen is shrunken, has an abnormal exine structure, and shows strong disorganization of internal membranes, particularly of the endoplasmic reticulum system.

## Summary

Rab proteins are post-translationally geranylgeranylated by Rab geranylgeranyl transferase (RGT) αβ. Mutations in each of the *RGTB* genes cause a tip growth defect whereas the double mutant is male sterile.

## Introduction

Eukaryotic cells are dependent on vesicular transport, employing protein machinery that is conserved throughout all Eukarya. Crucial players in this process are small GTPases, especially Rab proteins. Rabs possess a GTP-binding site and low intrinsic GTPase activity. In the yeast *Saccharomyces cerevisiae* the Rab protein family has only seven members, but in higher eukaryotes the diversification of Rab functions in specialized tissues and cell types results in as many as 57 Rabs in *Arabidopsis* and 60 in mammals (Pereira-Leal and Seabra, 2001). In plants the Rab family is divided into eight subfamilies, depending on the protein localization and general function in the cell (Rutherford and Moore, 2002).

All Rab proteins comprise a globular domain and an unstructured C-terminal tail of ~35 amino acids (Pfeffer, 2005). Structural motifs of the globular domain determine the Rab specificity towards interacting proteins. At the very end of the C-terminal tail lies a double cysteine motif, which serves as the target for geranylgeranylation by Rab geranylgeranyl transferase (RGT) (Rak *et al.*, 2004; Wu *et al.*, 2009). This modification with two 20-carbon isoprenoid chains enables anchoring of Rab proteins to membranes. A lack of this modification (due to mutations of these particular cysteines) has a disastrous effect on Rab function, since unprenylated Rabs are soluble and therefore cannot play their roles in vesicle formation, transport, and delivery (Gomes *et al.*, 2003).

RGT is a heterodimeric enzyme built of α and β subunits (RGTA and RGTB) (Guo *et al.*, 2008). The RGT substrate, a newly synthesized Rab protein, is initially recognized by Rab escort protein (REP; Rak *et al.*, 2004). REP escorts the Rab to the RGT heterodimer, where the two adjacent cysteines close to the C-terminus of Rab are prenylated (Guo *et al.*, 2008). Only the rat RGT has been crystallized so far (Pylypenko *et al.*, 2003). The lipid substrate-binding β subunit of this enzyme (RGTB) is highly conserved in all eukaryotes (Rasteiro and Pereira-Leal, 2007). It is a one-domain protein of α-barrel fold with a helical lid at the very C-terminus covering the entrance to the lipid-binding cavity (Pylypenko *et al.*, 2003). A model of the Rab prenylation reaction has been proposed recently (Guo *et al.*, 2008). In this model RGTB does not contribute substantially to either the recognition or the binding affinity of the Rab protein.

The yeast *S. cerevisiae* null mutant in the *RGTB* gene, *bet2*, is non-viable (Ferro-Novick *et al.*, 1991; Rossi *et al.*, 1991), similarly to mutants in the *RGTA* or *REP* genes (*bet4* and *mrs6*, respectively). A conditional mutant in the *RGTB* gene is thermosensitive for growth and accumulates endoplasmic reticulum (ER) membrane (Newman and Ferro-Novick, 1987). This mutant shows pleiotropic defects in protein transport at many different steps such as secretion to the periplasm and the vacuole or transport from the ER. Also the Rab proteins Ypt1 and Sec4 are mislocalized in this mutant (Rossi *et al.*, 1991).

Apart from yeast, effects of loss-of-function mutations in *RGTB* have been studied so far only in *Arabidopsis thaliana* (Hala *et al.*, 2010). Duplication of the *RGTB* gene has been found in several plant species, among them *Arabidopsis* (Rasteiro and Pereira-Leal, 2007). The *AtRGTB1* and *AtRGTB2* coding sequences are very similar to each other and to their mammalian homologues (56% identity for *AtRGTB1*) (Hala *et al.*, 2010). According to the Genevestigator database, the *AtRGTB1* transcript is abundant in all vegetative and generative tissues, and *AtRGTB2* mRNA is less abundant than *AtRGTB1* in the sporophyte, but is expressed at a similar level in pollen. Disruption of *AtRGTB1* disturbs the growth and development of the plant shoot (Hala *et al.*, 2010). The plants are dwarfed, show loss of apical dominance, and have abnormally developed flowers. The *Atrgtb1* mutants show a gravitropic defect of the shoot and do not etiolate in the dark. Deregulation of both exo- and endocytosis as well as accumulation of unprenylated Rab proteins in the cytosol of the mutant plant hypocotyls have been observed. The lack of AtRGTB1 protein results in the reduction of Rab geranylgeranylation activity in plant extracts towards RabA proteins to ~25% that of the wild type. The effect of *AtRGTB2* loss-of-function mutation has not been studied so far.

Rabs play vital roles in cells (Pfeffer, 2013). They recruit membrane-tethering and docking factors that facilitate membrane fusion (Hala *et al.*, 2008; Heider and Munson, 2012; Kim and Brandizzi, 2012), interact with motor proteins on transport vesicles, enable vesicle motility on actin cables (Voigt *et al.*, 2005; Samaj *et al.*, 2006; Pei *et al.*, 2012), and interact with enzymes of phosphatidylinositol metabolism modulating the membrane curvature and fluidity (Thole and Nielsen, 2008; Delage *et al.*, 2013). Moreover, Rabs define the compartment identity and gather integral and peripheral membrane proteins (e.g. lipid kinases) into specific domains on an organelle by regulating protein–lipid and protein–protein interactions (Cole and Fowler, 2006).

Importantly, the Rab-dependent vesicle trafﬁc is essential for establishing and maintaining polarity in plant cells, particularly those involved in tip growth (Voigt *et al.*, 2005; Samaj *et al.*, 2006; Cole and Fowler, 2006; Fu, 2010; Pei *et al.*, 2012). Root hairs and pollen tubes are polar tubular outgrowths of trichoblasts (root hair-forming epidermal root cells) and pollen grains, respectively, which grow exclusively at their tip. Rabs are highly abundant in tip-growing cells, and defects in their synthesis or function lead to a loss of cell polarity and defects of the cell shape (Cheung *et al.*, 2002; de Graaf *et al.*, 2005; Preuss *et al.*, 2006; Thole *et al.*, 2008; Camacho *et al.*, 2009; Szumlanski and Nielsen, 2009; Peng *et al.*, 2011).

Here a detailed phenotypic characterization of *A. thaliana* mutants in the two genes encoding the β subunit of Rab geranylgeranyl transferase, *Atrgtb1* and *Atrgtb2*, is provided. It is shown that the single mutants differ significantly from each other in flower morphology. Both *Atrgtb1* and *Atrgtb2* show affected polar growth of pollen tubes and root hairs. Finally, it is shown that double mutation of the two *AtRGTB* genes, completely abolishing Rab prenylation, results in absolute male sterility. This defect is due to impaired structure of membranes in the pollen grain and abnormal pollen exine formation.

## Materials and methods

### Plant material

*Arabidopsis thaliana* ecotype Columbia was used throughout the work. Insertion lines *Atrgtb1-1* SALK_015871 and *Atrgtb1-2* SALK_125416, *Atrgtb2-1* SALK_027208C and *Atrgtb2-2* SALK_149200 from the SALK Institute Collection were ordered from the Nottingham Arabidopsis Stock Center. Genotyping was performed with the primers designed by the T-primer design tool and LBb1.3 primer. The *Atrgtb1* lines were maintained as heterozygous segregating lines due to reduced fertility of the homozygote. Homozygous plants for further experiments were identified due to the characteristic phenotype and/or genotyping. The *Atrgtb2* lines were maintained as homozygous lines.

### Growth conditions and media

Plants were grown in a greenhouse under a long-day (16h light) photoperiod. Plants for RNA extraction and reverse transcription–PCR (RT–PCR) were grown in a hydroponic culture in Gilbert medium in a growth chamber (AR-66L CLF Plant Climatics). Seedlings for microscopic observations of root hairs were grown on vertical plates with half-strength Murashige and Skoog (MS) medium supplemented with vitamins and 1% sucrose, solidified with 1.2% agar. *In vitro* pollen germination and growth were performed on solid medium overnight (16h) at 22 °C according to Boavida and McCormick (2007).

### RNA extraction and RT–PCR

RNA was isolated from plant organs with the RNeasy Plant Mini Kit (Qiagen) according to the manufacturer’s protocol. After normalization of the amount of RNA, reverse transcription was carried out with an RT SuperScript II kit (Invitrogen) with an oligo(dT) primer. Semi-quantitative PCR to estimate the relative expression levels of *AtRGTB1* and *AtRGTB2* was carried out with the following pairs of primers: 5ʹ-AGCCTTGCGGCCATATTGTT-3ʹ and 5ʹ-CGAATTCATGAGCTCTACGTCTTCCTC-3ʹ for *AtRGTB1*, and 5’-GTCTAACTCCAACGCCTAAC-3ʹ and 5ʹ-CGAATTCGCG ATGGCAGACAAGCTCGT-3ʹ for *AtRGTB2*. Actin gene expression measured with the primer pair 5ʹ-ATTCGATCACTCAGA GCTAC-3ʹ and 5ʹ-AAGGAAGTACAGTGTCTGGA-3ʹ was used for normalization. All primers were designed with Clone Manager Suite 3 (SciEd Central) and synthesized at the Laboratory of DNA sequencing and oligonucleotide synthesis, IBB Warsaw, Poland (http://oligo.ibb.waw.pl/).

### Light microscopy

The morphology of flowers and siliques was studied under an SMZ1500 stereomicroscope (Nikon Instruments) equipped with a colour camera. Image acquisition was done with Lucia software (Laboratory Imaging). The morphology of root hairs and pollen germinating *in vitro* was observed under an Eclipse E800 microscope (Nikon Instruments) equipped with a CCD camera (Hamamatsu). Image acquisition was performed with Lucia software (Laboratory Imaging). At least 10 visual fields of material derived from 10 different plants obtained from three independent plant cultivations were used for scoring in each case (several hundreds individual root hairs/pollen tubes in total).

### *In vivo* pollen germination

Pollen germination *in vivo* was performed as follows: pistils of mature wt Col-0 flowers were prepared for manual pollination and 24h later were pollinated with appropriate pollen. After 16h, the pistils were treated with Alexander’s stain without fixation according to Cole *et al.* (2005) and Lalanne *et al.* (2004). Germination of pollen on the stigma was recorded under an inverted TE2000 microscope (Nikon Instruments). Image acquisition was performed with the use of a colour camera and NIS-Elements software (Nikon Instruments). At least 10 experiments were carried out for each line with pollen donors obtained from three independent plant cultivations.

### Staining of pollen anthers with Alexander stain

Mature anthers just before dehiscence were fixed in Carnoy fixative (60% ethanol, 30% chloroform, 10% glacial acetic acid) for 2h and stained with Alexander stain according to Lalanne *et al.* (2004). Anthers were observed under an inverted TE2000 microscope (Nikon Instruments). Image acquisition was performed with the use of a colour camera and NIS-Elements software (Nikon Instruments). Anthers obtained from at least five plants from two independent cultivations were observed.

### Confocal microscopy

Incorporation of FM 1–43 dye into growing root hairs was observed under a C1 confocal microscope (Nikon Instruments) with 488nm excitation from an argon ion laser and fluorescence detection with a 610LP emission filter. Roots of 9-day-old seedlings were transferred to 10 μM FM 1–43 solutions for 15min on ice and washed in a drop of water on a microscope slide. Observations of a single root hair were performed at the indicated time points. Images were recorded as *z*-stacks of ~20 optical sections with the EZ C1 image acquisition software (Nikon Instruments) and processed with EZ-C1 Viewer v.3.6 (Nikon Instruments). Experiments were performed on at least 10 roots from each mutant with at least three independent plant cultivations. Representative results are shown.

### Scanning electron microscopy (SEM) and transmission electron microscopy (TEM)

For TEM observations, flowers (green buds) were fixed in 2.5% glutaraldehyde in 100mM cacodylate buffer (pH 7.2) overnight, rinsed once in the buffer, and post-fixed in 1% osmium tetroxide overnight. Samples were rinsed, dehydrated in a graded ethanol series, and finally embedded in epoxy resin. Ultrathin sections were cut with a diamond knife on an MTX ultramicrotome (RMC Boeckeler Instruments). Specimens were examined using a LEO 912AB transmission electron microscope (Carl Zeiss). For SEM observations, pollen was spilled directly on microscope tables, coated with a thin layer of gold, and examined using a LEO 1430VP scanning electron microscope (Carl Zeiss).

### Statistical analysis

Statistical analysis was performed with the Graph Pad Prism program (Graphpad software). For analysis of trait inheritance, χ^2^ test against the appropriate H_0_ hypothesis, as described in Tables 1 and 2, was performed. In the case of features distributed bimodally, the exact binomial test was performed.

**Table 1.** Segregation of mutant alleles in the F_1_ generation of a self-cross of the *Atrgtb1+/–Atrgtb2–/–* *mutant* *Atrgtb1+/–Atrgtb2–/–* plants were self-crossed. Analysis of the genotypes in the F_1_ generation was performed by binomial exact test against the H_0_ hypothesis that one in four of the progeny carries a double mutation in *RGTB* genes.

Cross	*n*	Expected fraction of *rgtb1–/–rgtb2-/-*	Expected number of *rgtb1–/– rgtb2-/-*	Observed number of *rgtb1–/–rgtb2–/–*	*P*-value
*rgtb1-1+/–rgtb2-2-/–*×*rgtb1-1+/–rgtb2-2–/–*	82	1/4	20.5	0	5ʹ68884e-11***
*rgtb1-2+/–rgtb2-1–/–*×*rgtb1-2+/–rgtb2-1–/–*	128	1/4	32	0	1.01822e-16***

*n*, number of plants analysed.

***P-value <0.001.

**Table 2.** Segregation of mutant alleles in the F_1_ generation of the cross of pollen derived from *Atrgtb1+/–Atrgtb2–/–* on wt stigmaPollen derived from *Atrgtb1+/–Atrgtb2–/–* was crossed to wt stigma. Analysis of the genotypes in the F_1_ generation was performed by binomial exact test against the H_0_ hypothesis that segregation is Mendelian.

Cross	*n*	Expected fraction of *rgtb1+/–rgtb2+/–*	Expected number of *rgtb1+/–rgtb2+/–*	Observed number of *rgtb1+/–rgtb2+/–*	*P*-value
*rgtb1-1+/–rgtb2-2–/–*×wt stigma	21	1/2	11.5	0	4.76837e-07***
*rgtb1-2+/–rgtb2-1–/–*×wt stigma	21	1/2	11.5	0	4,76837e-07***

*n*, number of plants analysed.

***P-value <0.001.

The number of seeds per silique in mutant lines was compared with an appropriate experimental control. At least 20 siliques per plant line (wt or mutant) were used (4–5 plants obtained from at least two cultivations). Gaussian distribution of the obtained values was assumed. Statistical significance of the data was analysed by unpaired two-tailed Student t-test with Welch correction for inequality of variances.

The fraction (%) of abnormal pollen tubes/root hairs in mutant lines was compared with the wt. Statistical significance of the data was analysed by unpaired two-tailed Mann–Whitney test (due to the fact that the number of pollen tubes/root hairs analysed cannot be defined *a priori* and thus the fraction of abnormal pollen tubes/root hairs cannot be assumed to have a Gaussian distribution, the non-parametric test was chosen for analysis).

### Amino acid and nucleotide sequence analysis and insert positioning

*AtRGTB1* and *AtRGTB2* nucleotide sequences, NM_121259 and NM_180236, respectively, were derived from the EMBL server. Corresponding amino acid sequences, NP_568259 and NP_850567, respectively, were derived from the PDB server. Alignments were performed with the T-coffee (http://www.ebi.ac.uk/Tools/msa/tcoffee) and visualized with Jalview. DNA obtained from genotyping PCR reaction of each mutant line was sequenced from the Lbb1.3 primer (http://oligo.ibb.waw.pl/). Results of the sequencing were aligned with genomic sequence of the *AtRGTB1* and *AtRGTB2* genes with the EMBOSS program (http://emboss.bioinformatics.nl).

## Results

### Characterization of *AtRGTB2*

The *AtRGTB2* gene is located on the third chromosome, comprises nine exons, and its structure is very similar to that of its paralogue, *AtRGTB1*, except that the sequence corresponding to the first exon of *AtRGTB2* is split into two shorter exons in the *AtRGTB1* gene (Supplementary Fig. S1 available at *JXB* online). The nucleotide and protein sequences of *AtRGTB1* and *AtRGTB2* are very similar to each other, showing ~87% nucleotide identity and 94% amino acid similarity (Supplementary Fig. S1). To study the effects of single-gene disruptions of *AtRGTB1* or *AtRGTB2* on plant development, two different insertion mutant lines for each gene were chosen. The location of the insert was confirmed for the former (Hala *et al.*, 2010) and determined for the latter pair of mutants (Supplementary Fig. S1).

**Fig. 1.**
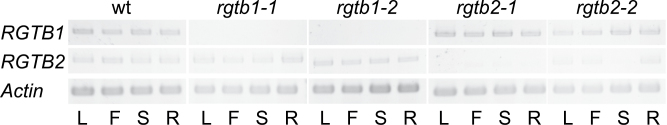
Expression of *AtRGTB1* and *AtRGTB2* in *Atrgtb* homozygous lines. Semi-quantitative RT–PCR in different organs of plants. L, leaf; F, flower; S, stem; R, root. Actin mRNA was used as normalization control. Results representative of three independent plant cultures are shown.

The level of expression of both genes in different organs of plants grown in a hydroponic culture was analysed using semi-quantitative RT–PCR. In both *Atrgtb1* homozygotes, the *AtRGTB1* mRNA was undetectable, while it was present at a high level and ubiquitous in the wt and the two *Atrgtb2* lines (Fig. 1). Consistently, the expression of the *AtRGTB2* gene was down-regulated in the *Atrgtb2-1* line, on contrast to the expression of the possibly truncated *AtRGTB2* mRNA detectable in the *Atrgtb2-2* line (this truncation may lead to partially active protein, since it probably does not destroy the overall structure of the α-barrel). The expression of *AtRGTB2* in wt and *Atrgtb1* homozygotes was low, but ubiquitous. Up-regulation of *AtRGTB2* in *Atrgtb1* homozygotes could not be observed by this experimental method.

To summarize, the results regarding *AtRGTB* expression confirmed the data from the Genvestigator database referred to by Hala *et al.* (2010) and those from the Bio-Array Resource website (BAR, http://bar.utoronto.ca; Toufighi *et al.*, 2005) and were in line with the severity of the phenotypes observed in the *Atrgtb1* and *Atrgtb2* homozygotes (see below). *Atrgtb1* plants were strongly affected, forming small rosettes and a low, branched stem with malformed flowers, and they were delayed in flowering time (Hala *et al.*, 2010). In contrast, *Atrgtb2* plants were indistinguishable from the wt plants in their morphology and the time of flowering (Supplementary Fig. S2 at *JXB* online).

### Morphology of generative organs in *Atrgtb* mutants

**Fig. 2.**
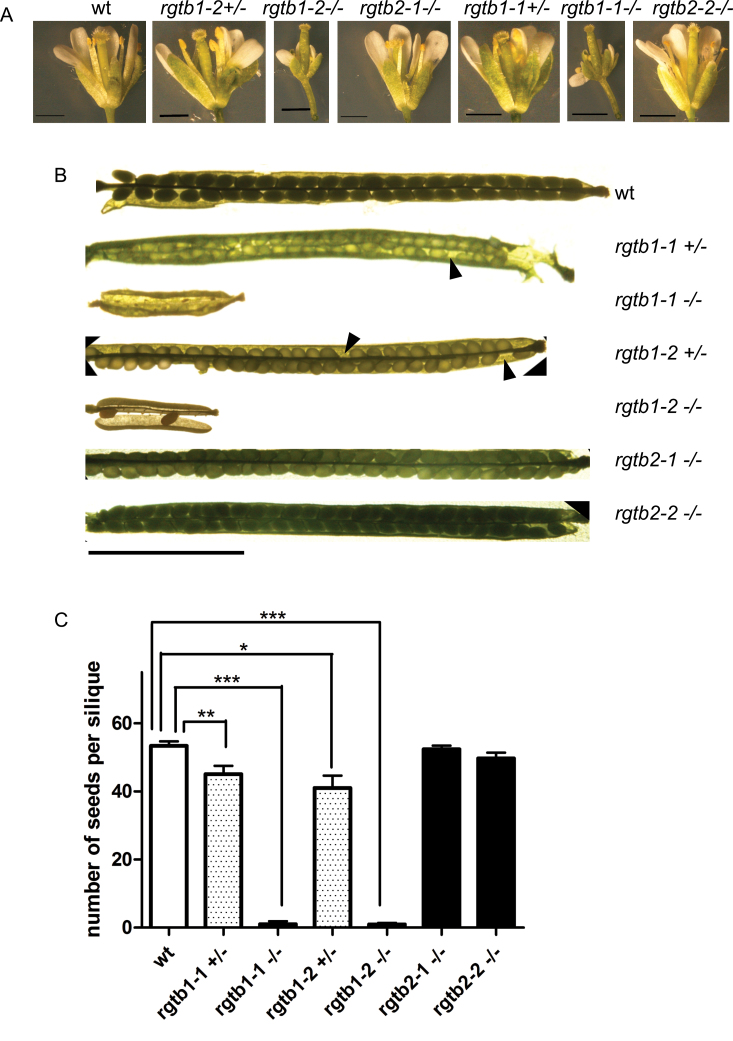
Morphology of generative organs in *Atrgtb* mutants. (A) Flowers, scale bar=1mm. (B) Siliques, scale bar=5mm; the arrowheads indicate the unfertilized ovules. (C) Number of seeds per silique after self-pollination, *n*>20. Bars represent the mean ±SE. Statistical significance analysed by Student *t*-test, *P<0.05, **P<0.01, ***P<0.001.

Hala *et al.* (2010) described that *Atrgtb1* mutants have malformed flowers with small stamens developing much later than pistils. In contrast, this kind of deformation was not found in any of the homozygous *Atrgtb2* lines investigated (Fig. 2A). A closer inspection of the generative organs of both *Atrgtb1* and *Atrgtb2* mutants drew attention to the possibility that the self-infertility of the *Atrgtb1* mutants was not due solely to disturbed flower development. Siliques of homozygous *Atrgtb1* mutants were smaller and rarely contained any seeds, while both homozygous *Atrgtb2* mutants produced normal siliques (Fig. 2B, C). This observation was interesting, since the pollen of *Atrgtb1* mutants was able to pollinate wt stigma, and wt pollen could pollinate *Atrgtb1* stigma (reciprocal crosses were succesful; see below). The *Atrgtb1* pistils protruded from petals in a mature flower ready for pollination (Fig. 2; Hala *et al.*, 2010). Thus, such flowers could not be fertilized by pollen from the same flower due to the reduced height of the stamens but could easily be pollinated by pollen from adjacent flowers from the same plant. Such pollination, however, happened only occasionally and led to the formation of very few seeds per silique (Fig. 2C). Plants grown from such seeds morphologically resembled the parent and were genotyped to be *Atrgtb1*. This observation suggested that, apart from the flower deformation, other reasons, related to gametophyte development, pollen tube growth, or fertilization ability, may contribute to the very low *Atrgtb1* self-fertility.

The number of *Atrgtb1–/–* progeny in the F_1_ generation after self-pollination of an *Atrgtb1+/–* heterozygote was not considerably lower (*P* =0.065 for *Atrgtb1-2+/–* and *P*=0.2459 for *Atrgtb1-1+/–* by the χ^2^ test) than the fraction expected from Mendelian segregation, and for the *Atrgtb2+/–* mutant the corresponding fraction was exactly as expected from Mendelian segregation.

### Pollen germination and tube growth are impaired in *Atrgtb* mutants

**Fig. 3.**
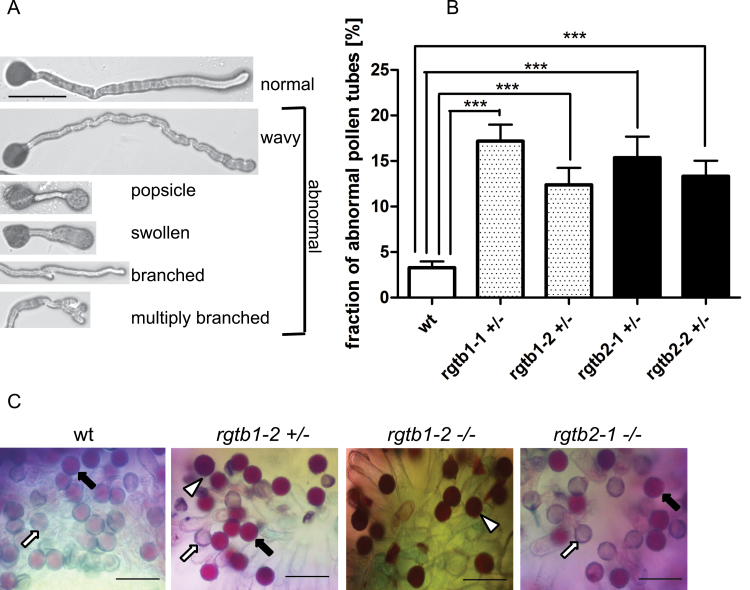
Male gametophyte morphology in *Atrgtb* mutants. (A) Morphological aberrations in pollen tubes germinated *in vitro*; scale bar=100 μm. (B) Frequency of pollen tube abnormalities; bars represent the mean ±SE. Statistical significance was analysed by Mann–Whitney test, ***P<0.001. (C) *In vivo* germination of mutant pollen on wt stigma. Alexander stain 16h after pollination; grey and pale pink pollen grains are germinated (arrows), dark purple grains are non-germinated (white arrowhead). Scale bar=100 μm. Note shrunken pollen grains deposited on stigma for *Atrgtb1+/–* mutant pollen (black arrowhead) and low frequency of germination for *Atrgtb1–/–* pollen. Representative images are shown.

Pollen germination and pollen tube growth *in vitro* were studied on solid germination medium. After 4h or 16h, growing pollen tubes were observed under a light microscope. Pollen derived from *Atrgtb1-1–/–* or *Atrgtb1-2–/–* plants hardly germinated in these conditions. For this reason, the germination of pollen derived from heterozygous plants was studied, always in parallel to wt-derived pollen. After 4h, the emerging pollen tubes were very short and in many cases germination had not started at all; therefore, the quantitative observations were performed after 16h. Pollen derived from *Atrgtb1+/–* plants germinated well, but the ratio of abnormally developing pollen tubes (tip swelling or branching) was significantly higher than in wt pollen (Fig. 3A, B). Similarly, pollen derived from *Atrgtb2+/–* plants (also heterozygous to enable easier comparison) showed swelling and shortening of pollen tubes. Branched pollen tubes were more frequently found in the pollen derived from *Atrgtb1+/–* than in that derived from *Atrgtb2+/–*, but the difference was not statistically significant.

To complement *in vitro* observations, *in vivo* pollen germination was studied. The method allows non-germinated grains to be distinguished from those with growing pollen tubes. Approximately 16h after pollination, most pollen grains derived from wt and *Atrgtb2-1–/–* plants had already emptied (grey grains in Fig. 3C, white arrow) or started germination (pink pollen grains in Fig. 3C, black arrow). Pollen derived from *Atrgtb1-2–/–* was scarce and always stained dark purple, which means it had not germinated, but after 24h growing pollen tubes or emptied pollen grains were occasionally observed (Fig. 3C, non-germinating pollen indicated by white arrowhead). Interestingly, many grains of pollen derived from *Atrgtb1-2+/–* also stained dark purple (Fig. 3C, white arrowhead). However, in the case of *Atrgtb1-2+/–* and, to a lesser extent, *Atrgtb1-2–/–*, a fraction of shrunken grains deposited on a stigma never formed pollen tubes (Fig. 3C, black arrowhead). These *in vivo* observations, although only qualitative, indicate that in the *Atrgtb1* mutant both production and germination of pollen may be defective.

### Root hair morphology is altered in *Atrgtb* mutants

**Fig. 4.**
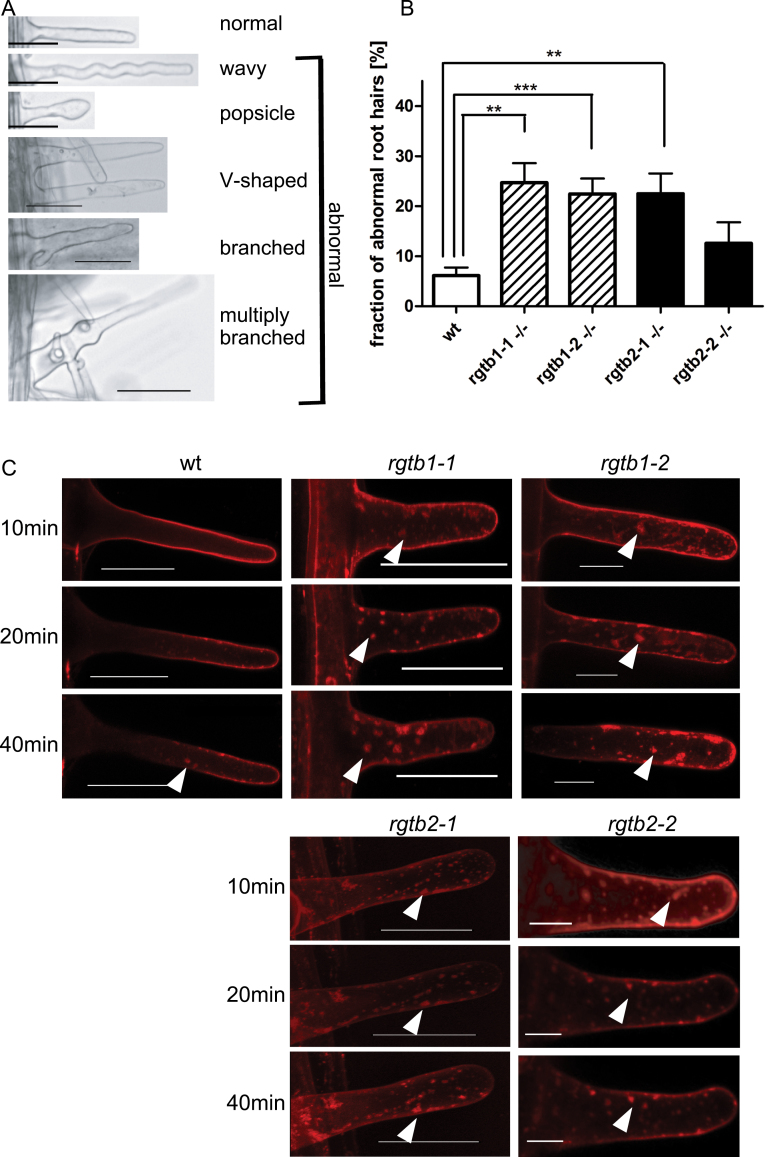
Root hair morphology and functioning in *Atrgtb* mutants. (A) Aberrations in root hair morphology; scale bar=30 μm. (B) Frequency of abnormal root hairs; bars represent the mean ±SE. Statistical significance was analysed by Mann–Whitney test, **P<0.01, ***P<0.001. (C) Time course of FM 1-43 styryl dye uptake and recycling in root hairs, confocal microscopy. *Z*-stacks of ~20 images from each root hair are shown. Scale bar=30 μm. Note large dye aggregates in mutant cells (arrowhead). Representative images are shown.

Since tip growth is a developmental feature specific for pollen tubes and root hairs, the morphology of the latter cells in the *Atrgtb1* and *Atrgtb2* mutants was examined. The overall morphology of roots of the *Atrgtb1* and *Atrgtb2* mutants seemed normal (Supplementary Fig. S2 at *JXB* online; see also Hala *et al.*, 2010). However, the frequency of abnormal, branched (sometimes even multiply) or swollen, root hairs was significantly higher for both homozygous mutants in comparison with wt plants (Fig. 4A, B). The branching was more frequent in *Atrgtb1* plants than in *Atrgtb2*, but the difference was not statistically significant. The disturbed root hair development indicates that the functioning of these cells is compromised by disruption of either *AtRGTB* gene.

To test the root hair functionality, endocytosis and membrane recycling were studied in these cells by following the incorporation of a styryl dye (Ovecka *et al.*, 2008). In wt plants the FM 1–43 dye was incorporated into the outer membrane of the cell within ~10min and later small vesicles in the cytoplasm became fluorescent (40min) (Fig. 4C), in agreement with earlier observations (Ovecka *et al.*, 2008; Geldner *et al.*, 2009). However, even in wt root hairs, the accumulation of vesicles in the so-called ‘inverted cone’ region in short time frames was rarely observed here, probably due to different experimental conditions compared with those of Ovecka *et al.* (2008). For both *Atrgtb1* and *Atrgtb2* mutants, the process of large vesicle formation was much faster and the staining more intense: large vesicles were visible in the cytoplasm within 10–20min (Fig. 4C). This result combined with the earlier findings regarding pollen tube growth showed that cells known to carry out intense vesicular transport (pollen tubes and root hairs) are affected by both *AtRGTB2* and *AtRGTB1* mutations. From the results presented it might be concluded that in the root hair these mutations have a stronger effect on exocytosis and vesicle recycling than on endocytosis.

### *Atrgtb1Atrgtb2* double mutation is pollen-lethal in *Arabidopsis*

Finally, an effort was made to construct a homozygous double mutant in both *AtRGTB* genes. To this end several independent crosses were set of selected *Atrgtb1* mutants (homozygotes or heterozygotes) with *Atrgtb2* homozygotes. In the F_2_ generation, the expected Mendelian ratio of inheritance of the double mutation is 1/16. Two hundred *Atrgtb1-1*×*Atrgtb2-2* and 202 *Atrgtb1-2*×*Atrgtb2-1* F_2_ progeny plants were analysed, but no double mutant homozygote was found, although 13 were expected assuming Mendelian inheritance.

**Fig. 5.**
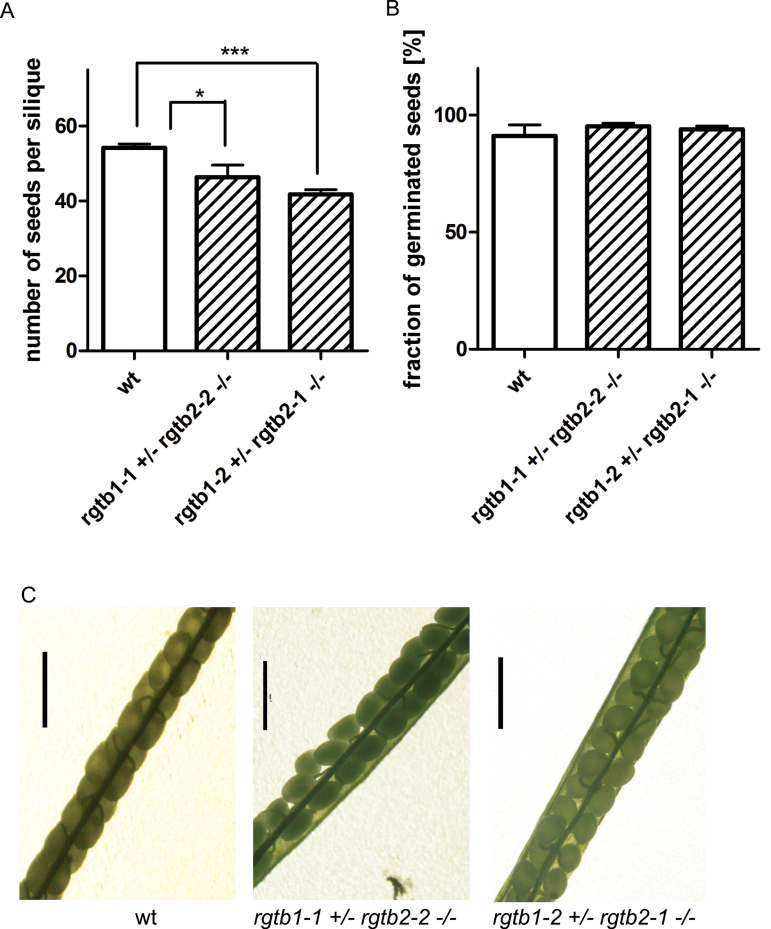
Male gametophyte defect in the *Atrgtb1Atrgtb2* double mutant. (A) Number of seeds per silique, *n*>20; bars represent mean ±SE. (B) Frequency of germination of seeds from self-pollination of *Atrgtb1+/–Atrgtb2–/–* plants, *n*>250. Statistical significance analysed by Student *t*-test, *P<0.05, ***P<0.001. (C) Siliques of an *Atrgtb1+/–Atrgtb2–/–* plant after self-pollination; scale bar=1mm. (This figure is available in colour at *JXB* online.)

To verify the assumption that the lack of RGT activity is lethal in plants and to investigate the stage on which lethality occurs, genotyping of plants from the F_3_ generation of the initial crosses, namely the progeny of *Atrgtb1+/–Atrgtb2–/–* self-pollination, was performed. In F_3_ progeny of both initial crosses no double mutant homozygotes were found, although 1:2:1 segregation was assumed (Table 1). To check whether the double *Atrgtb1–/–Atrgtb2–/–* mutation causes embryolethality, the siliques of the *Atrgtb1+/–Atrgtb2–/–* plants after self-pollination were opened. The number of seeds was only slightly diminished compared with the wt, with a surprisingly low number of unfertilized ovules or dead embryos (Fig. 5). Moreover, the ratio of seed germination and the lethality of young 1-week-old seedlings were the same as in the wt in both F_3_ progeny lines (Fig. 5A, B).

This observation indicated that the double *Atrgtb1–Atrgtb2–* mutation causes lethality already at the stage of the male gametophyte. To verify that assumption further, wt stigmas were pollinated with pollen derived from *Atrgtb1+/–Atrgtb2–/–*; again no *Atrgtb1+/–Atrgtb2+/–* progeny was found. This observation confirmed that *Atrgtb1–Atrgtb2–* is pollen-lethal (Table 2).

### *Atrgtb* mutants pollen development and ultrastructure

**Fig. 6.**
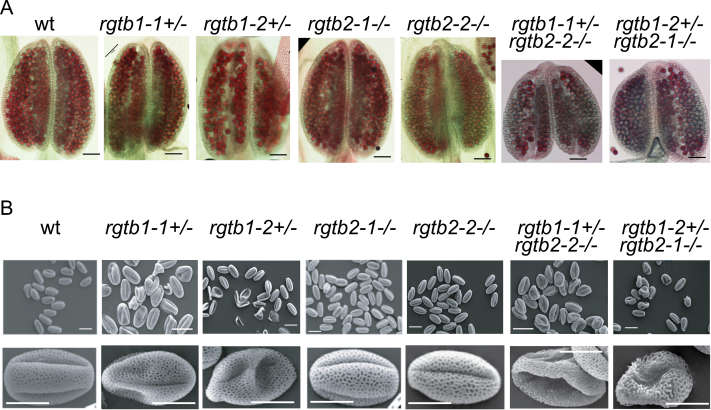
Pollen grain development in single mutants in *AtRGTB* and in the *Atrgtb1Atrgtb2* double mutant. (A) Alexander stain of mature anthers; scale bar=100 μm. (B) SEM images of mature pollen grains. Note that in pollen derived from *Atrgtb1+/–* some grains are hollow, but of normal length, but in pollen derived from *Atrgtb1+/–Atrgtb2–/–*, half of the grains are shrunken and many of them show abnormal exine structure; scale bar=10 μm for higher magnification and 20 μm for lower magnification. Representative images are shown. (This figure is available in colour at *JXB* online.)

To check whether *Atrgtb1–Atrgtb2–* pollen is produced, the mature anthers of the wt and both lines of *Atrgtb1-1+/–Atrgtb2-2–/–* and *Atrgtb1-2+/–Atrgtb2-1–/–-* plants were stained with Alexander stain to visualize viable pollen grains. Wt and *Atrgtb2* anthers were full of pollen grains. In other cases, empty spaces between pollen grains were visible, meaning, that not all pollen grains developed correctly (Fig. 6A). Direct visualization of pollen grains was performed with SEM (Fig. 6B). Again, all pollen grains from wt and *Atrgtb2–/–* plants were of normal size, showing characteristic regular exine structure. Pollen grains from *Atrgtb1+/–* plants were of normal length, but a few of them were hollow; however, exine structure was regular. About half of the pollen grains derived from *Atrgtb1+/–Atrgtb2–/–* were shrunken, many of them showing irregular exine structure.

**Fig. 7.**
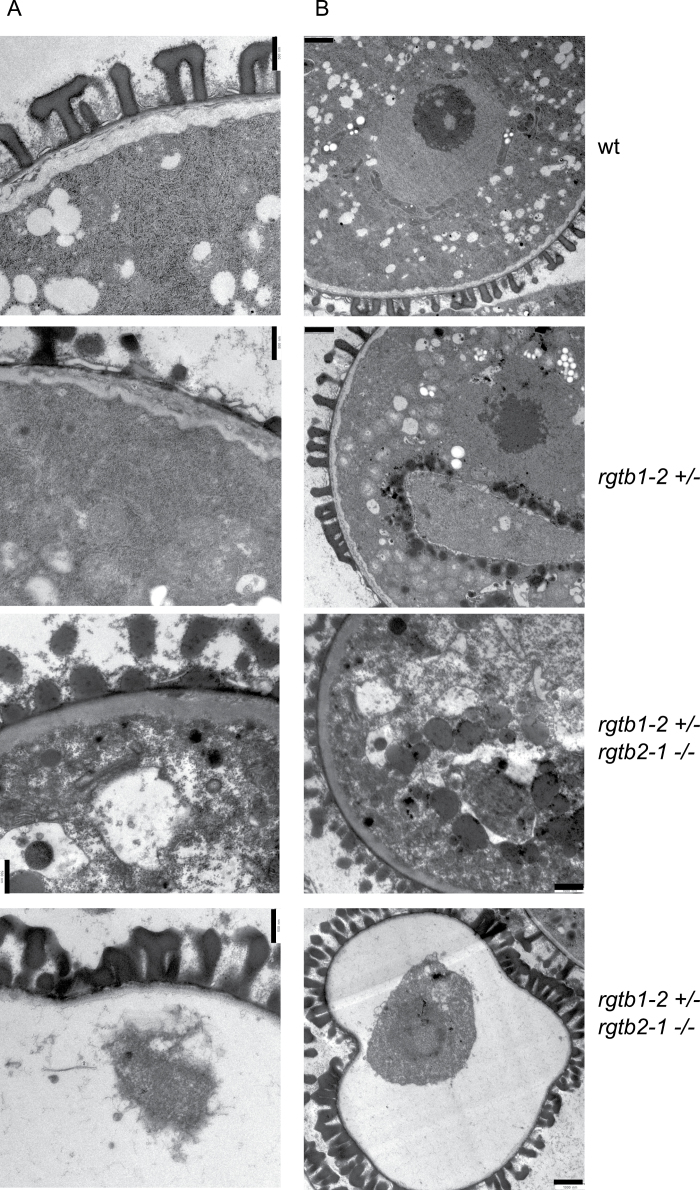
Bicellular pollen grain ultrastructure; ultrathin section TEM images of wt, *Atrgtb1–* and *Atrgtb1–Atrgtb2–* pollen. (A) Cell wall and exine ultrastructure; scale bar=500nm. (B) General view of a pollen grain; scale bar=1 μm. Representative images are shown.

To characterize *Atrgtb1–Atrgtb2–* pollen further, TEM analysis was conducted on selected lines, namely the wt and *Atrgtb1-2+/–* treated as the control, and the *Atrgtb1-2+/–Atrgtb2-1–/–* double mutant. Pollen from the *Atrgtb2-1–/–* line was indistinguishable from that of the wt and was therefore not included in the analysis. In contrast pollen derived from the *Atrgtb1-2–/–* line showed additional effects, probably of maternal (tapetum) origin, that were not visible in pollen derived from *Atrgtb1-1+/–* (data not shown) and therefore could not serve as an appropriate control. Immature pollen derived from closed green buds was studied (Fig. 7A, B). A high number of small vacuoles in the cytoplasm of pollen grains, the presence of two nuclei, and lack of degradation of tapetum cells surrounding the loculus in all observed specimens indicated the bicellular stage of pollen development (Quilichini *et al.*, 2014). At this stage, wt pollen showed a uniformly structured cell wall with clearly visible exine structures (T-shaped baculae and tecta). Undisturbed membrane structures were seen inside the cell, such as rough and smooth ER, the nuclear envelope, the plasma membrane, and the tonoplast. The structure of mitochondria and plastids was also unchanged. The ultrastructure of *Atrgtb1-2-* pollen derived from heterozygous *Atrgtb1-2+/–* plants was not noticeably changed. The only difference was that the cell wall was not so uniform, showing thinner and thicker sections, as well as intrusions into the cytoplasm. Some of the grains had underdeveloped exine structures and a few were hollow, with cytoplasm remnants in the middle of the grain (data not shown). *Atrgtb1–Atrgtb2–* pollen grains showed looser cytoplasmic structure, occasionally leading to pollen shrinkage. The vacuoles were larger and were not round shaped as in the wt; no rough or smooth ER was visible. Many of the more strongly affected grains imploded during the preparation procedures, and remnants of cytoplasm and nuclei were visible in them. It must be emphasized here that in each studied anther derived from *Atrgtb1+/–Atrgtb2–/–* plants, two kinds of pollen grains were visible at a time, some of them normal, some shrunken, so the changes in the cytoplasm are most probably not an artefact of the preparation methods.

To summarize, double mutation in *AtRGTB* genes most probably leads to a pollen development defect and this explains the lack of functional pollen in genetic crosses and lack of a double mutant in sporophytic generation of *Arabidopsis.*

## Discussion

The aim of this study was to elucidate the effects of disruption of Rab function in plants. To this end, the morphology and development of mutant *Arabidopsis* lines with disruption of *AtRGTB1* and/or *AtRGTB2* genes were analysed. Disturbed tip growth was the major effect of partial RGTB deficiency, while total RGTB deficiency led to absolute male sterility owing to a compromised pollen grain development.

The tip growth of cells is an evolutionarily ancient process. Exocytosis is required near the growing tip to provide new portions of plasma membrane components and the material for the new cell wall. Endocytosis and membrane cycling must be coupled with exocytosis to remove an excess of the plasma membrane from the tip (Cole and Fowler, 2006; Samaj *et al.*, 2006; Cheung and Wu, 2008; Lycett, 2008; Zhang and McCormick, 2010; Pei *et al.*, 2012). Functional, membrane-anchored Rabs are engaged in all these processes (Pfeffer, 2013). It is not surprising, therefore, that disturbing the enzymatic activity of AtRGT, the enzyme modifying all but one (AtRabF1) of the *Arabidopsis* Rabs (Ueda *et al.*, 2001), is disastrous for the plant.

Experiments presented in this work focus on the function of AtRGTB in the polar growth of plant cells. The best studied cells that show tip growth are root hairs and pollen tubes. The initiation, emergence, and growth of the root hair are strongly dependent on intracellular transport. Mutations affecting the transport and polarity-related proteins have been shown to disturb the process. In particular, a lack of an AtRabA4b effector, phosphatidylinositol-4 kinase (AtPI4Kβ; Preuss *et al.*, 2004, 2006; Thole *et al.*, 2008; Stenzel *et al.*, 2008; Camacho *et al.*, 2009) causes an abnormal growth and branching of root hairs. The types and distribution of the phenotypes described (Preuss *et al.*, 2006) correspond well to the fraction of aberrant root hairs observed in this study in *Atrgtb1* and *Atrgtb2* mutants. Mutants in the Rab-interacting exocyst complex (*Atexo70A1*), responsible for correct docking of the exocytosis vesicles to the plasma membrane, show root hair branching as well (Synek *et al.*, 2006). Studies on mutants lacking single Rab activity have not revealed such phenotypes, probably due to a high level of redundancy among various Rabs in plants.

Analyses of the incorporation of widely used ﬂuorescent styryl dyes, FM1-43 and FM4-64, have proved that endocytosis is tightly coupled with secretion in root hair cells and that vesicles undergo fusion with each other as well as with the plasma membrane (Ovecka *et al.*, 2008). The staining pattern observed for *Atrgtb1* and *Atrgtb2* mutants might be interpreted as slowing down of membrane recycling and exocytosis due to a lower amount of prenylated Rabs (Ovecka *et al.*, 2008). It is not clear which particular Rabs are responsible for this effect; however, it seems reasonable to assume that it is the hypoprenylated Rabs from the RabA family known to be engaged in endosome/*trans*-Golgi network recycling which are resposible. Hala *et al.* (2010) observed a decreased tempo of both exo- and endocytosis in hypocotyls of an *Atrgtb1* mutant; however, in contrast to observations presented in this study, they did not notice any changes in these processes in the root. This discrepancy could be due to this study focusing on one particular type of specialized cells, the root hairs. They are known to be highly active in transport and their membrane trafficking activity could therefore be much higher than the overall activity of other root cells and therefore more sensitive to any disturbances.

Similarly to root hairs, mutations in Rab-encoding genes have been reported to cause cell deformations in pollen tubes. *AtrabA4d* causes cell tip swelling and meandering (Szumlanski and Nielsen, 2009), and the double mutant *AtrabD2b/c* also produces abnormal pollen with swollen and occasionally branched tips (Peng *et al.*, 2011). Similar observations were made for tobacco pollen tubes (Cheung *et al.*, 2002; de Graaf *et al.*, 2005). The data presented here corroborate the earlier observations on single *Atrab* mutants. The *Atrgtb1–Atrgtb2–* double mutant pollen was non-viable, as was shown by studying genotype segregation in the F_2_ and F_3_ generations of the *Atrgtb1*×*Atrgtb2* cross and the reciprocal cross to wt stigma. Observations of siliques and seed germination excluded the possibility of embryolethality or seedling lethality of the double mutant. The pollen lethality of the double *RGTB* mutant turned out to be caused by disorganization of internal membrane structures, in particular the ER.

Accordingly, mutations in other genes coding for Rab effectors engaged in exocytosis cause similar pollen defects. Insertion mutants in *Arabidopsis* phosphatidylinositol-4-phosphate 5-kinases 4 and 5 cause reduced pollen germination and defects in pollen tube elongation. Overexpression of either of these kinases in *Arabidopsis* leads to a zigzag growth pattern or multiple branching of the pollen tubes, similar to that observed for *Atrgtb* mutants (Ischebeck *et al.*, 2008). Another good example is the effect of mutations in the exocyst complex subunits *Atexo70A1* and *Atsec5*, *6*, *8*, *15* (Cole *et al.*, 2005; Hala *et al.*, 2008). In these cases, mutant pollen causes a male-transmitted defect. Further, knock-out mutations in SNARE proteins (responsible for vesicle fusion) are lethal at the male gametophyte stage (Shirakawa *et al.*, 2010; Uemura *et al.*, 2012). Also, in this work, the single *Atrgtb* mutants showed abnormal tip growth and the double-mutated pollen was sterile.

A complete lack of Rab prenylation is lethal for a multicellular organism. as was shown here for an angiosperm plant and recently for the moss *Physcomitrella patens* (Thole *et al.*, 2014), where, similarly to *Arabidopsis*, two copies of the *RGTB* gene exist. Single mutants in its haploid sporophyte do not show a phenotypic defect, but the double mutant is lethal. Similarly, knock-outs in *RGTA* and *REP*, single*-*copy genes in *P. patens*, are lethal (Thole *et al.*, 2014). Accumulated RGT, Rabs, or their encoding mRNAs could be sufficient for the development of the *Atrgtb1–Atrgtb2–* female gametophyte in heterozygous *Arabidopsis* plants. The male gametophyte is, however, much more dependent on vesicular transport than is the mobile animal sperm, animal egg (Moosajee *et al.*, 2009), or the non-expanding female gametophyte, and is less dependent on nutrients and hormones secreted by surrounding sporophytic tissues.

In line with the observations from *P. patens*, the functions of *RGTB1* and *RGTB2* in *Arabidopsis* male haploid generation seem redundant due to a similar level of expression of these two genes in pollen (Genvestigator data referred to in Hala *et al.*, 2010). In contrast, in sporophytic tissues, the severe growth defects may be caused by insufficient dosage of RGTB rather than by distinct functions of RGTB1 and RGTB2 in the cells. This hypothesis awaits confirmation.

## Supplementary data

Supplementary data are available at *JXB* online.

Figure S1. *RGTB* gene structure, chromosome location, and nucleotide and amino acid alignment.

Figure S2. General morphology of seedlings, rosettes, shoots, and roots of *Atrgtb1* and *Atrgtb2* mutant homozygotes in comparison with wt Col-0 plants.

Supplementary Data

## References

[CIT0001] Boavida LC McCormick S 2007 Temperature as a determinant factor for increased and reproducible *in vitro* pollen germination in Arabidopsis thaliana. The Plant Journal 52, 570–582. 17764500 10.1111/j.1365-313X.2007.03248.x

[CIT0002] Camacho L Smertenko AP Perez-Gomez J Hussey PJ Moore I 2009 Arabidopsis Rab-E GTPases exhibit a novel interaction with a plasma-membrane phosphatidylinositol-4-phosphate 5-kinase. Journal of Cell Science 122, 4383–4392. 19903693 10.1242/jcs.053488

[CIT0003] Cheung AY Chen CY Glaven RH de Graaf BH Vidali L Hepler PK Wu HM 2002 Rab2 GTPase regulates vesicle trafficking between the endoplasmic reticulum and the Golgi bodies and is important to pollen tube growth. The Plant Cell 14, 945–962. 11971147 10.1105/tpc.000836 PMC150694

[CIT0004] Cheung AY Wu HM 2008 Structural and signaling networks for the polar cell growth machinery in pollen tubes. Annual Review of Plant Biology 59, 547–572. 10.1146/annurev.arplant.59.032607.092921 18444907

[CIT0005] Cole RA Fowler JE 2006 Polarized growth: maintaining focus on the tip. Current Opinion in Plant Biology 9, 579–588. 17010659 10.1016/j.pbi.2006.09.014

[CIT0006] Cole RA Synek L Zarsky V Fowler JE 2005 SEC8, a subunit of the putative Arabidopsis exocyst complex, facilitates pollen germination and competitive pollen tube growth. Plant Physiology 138, 2005–2018. 16040664 10.1104/pp.105.062273 PMC1183391

[CIT0007] de Graaf BH Cheung AY Andreyeva T Levasseur K Kieliszewski M Wu HM 2005 Rab11 GTPase-regulated membrane trafficking is crucial for tip-focused pollen tube growth in tobacco. The Plant Cell 17, 2564–2579. 16100336 10.1105/tpc.105.033183 PMC1197435

[CIT0008] Delage E Puyaubert J Zachowski A Ruelland E 2013 Signal transduction pathways involving phosphatidylinositol 4-phosphate and phosphatidylinositol 4,5-bisphosphate: convergences and divergences among eukaryotic kingdoms. Progress in Lipid Research 52, 1–14. 22981911 10.1016/j.plipres.2012.08.003

[CIT0009] Ferro-Novick S Newman AP Groesch M Ruohola H Rossi G Graf J Shim J 1991 An analysis of BET1, BET2, and BOS1. Three factors mediating ER to Golgi transport in yeast. Cell Biophysics 19, 25–33. 1726885 10.1007/BF02989876

[CIT0010] Fu Y 2010 The actin cytoskeleton and signaling network during pollen tube tip growth. Journal of Integrative Plant Biology 52, 131–137. 20377675 10.1111/j.1744-7909.2010.00922.x

[CIT0011] Geldner N Denervaud-Tendon V Hyman DL Mayer U Stierhof YD Chory J 2009 Rapid, combinatorial analysis of membrane compartments in intact plants with a multicolor marker set. The Plant Journal 59, 169–178. 19309456 10.1111/j.1365-313X.2009.03851.x PMC4854200

[CIT0012] Gomes AQ Ali BR Ramalho JS Godfrey RF Barral DC Hume AN Seabra MC 2003 Membrane targeting of Rab GTPases is influenced by the prenylation motif. Molecular Biology of the Cell 14, 1882–1899. 12802062 10.1091/mbc.E02-10-0639 PMC165084

[CIT0013] Guo Z Wu YW Das D 2008 Structures of RabGGTase-substrate/product complexes provide insights into the evolution of protein prenylation. EMBO Journal 27, 2444–2456. 18756270 10.1038/emboj.2008.164 PMC2543052

[CIT0014] Hala M Cole R Synek L 2008 An exocyst complex functions in plant cell growth in Arabidopsis and tobacco. The Plant Cell 20, 1330–1345. 18492870 10.1105/tpc.108.059105 PMC2438459

[CIT0015] Hala M Soukupova H Synek L Zarsky V 2010 Arabidopsis RAB geranylgeranyl transferase beta-subunit mutant is constitutively photomorphogenic, and has shoot growth and gravitropic defects. The Plant Journal 62, 615–627. 20180921 10.1111/j.1365-313X.2010.04172.x

[CIT0016] Heider MR Munson M 2012 Exorcising the exocyst complex. Traffic 13, 898–907. 22420621 10.1111/j.1600-0854.2012.01353.x PMC3374049

[CIT0017] Ischebeck T Stenzel I Heilmann I 2008 Type B phosphatidylinositol-4-phosphate 5-kinases mediate Arabidopsis and Nicotiana tabacum pollen tube growth by regulating apical pectin secretion. The Plant Cell 20, 3312–3330. 19060112 10.1105/tpc.108.059568 PMC2630452

[CIT0018] Kim SJ Brandizzi F 2012 News and views into the SNARE complexity in Arabidopsis. Frontiers in Plant Science 3, 28. 23018380 10.3389/fpls.2012.00028 PMC3355637

[CIT0019] Lalanne E Honys D Johnson A Borner GH Lilley KS Dupree P Grossniklaus U Twell D 2004 SETH1 and SETH2, two components of the glycosylphosphatidylinositol anchor biosynthetic pathway, are required for pollen germination and tube growth in Arabidopsis. The Plant Cell 16, 229–240. 14671020 10.1105/tpc.014407 PMC301407

[CIT0020] Lycett G 2008 The role of Rab GTPases in cell wall metabolism. Journal of Experimental Botany 59, 4061–4074. 18945942 10.1093/jxb/ern255

[CIT0021] Moosajee M Tulloch M Baron RA Gregory-Evans CY Pereira-Leal JB Seabra MC 2009 Single choroideremia gene in nonmammalian vertebrates explains early embryonic lethality of the zebrafish model of choroideremia. Investigative Ophthalmology and Visual Science 50, 3009–3016. 19117920 10.1167/iovs.08-2755

[CIT0022] Newman AP Ferro-Novick S 1987 Characterization of new mutants in the early part of the yeast secretory pathway isolated by a [3H]mannose suicide selection. Journal of Cell Biology 105, 1587–1594. 3312234 10.1083/jcb.105.4.1587 PMC2114650

[CIT0023] Ovecka M Baluska F Lichtscheidl I 2008 Non-invasive microscopy of tip-growing root hairs as a tool for study of dynamic and cytoskeleton-based vesicle trafficking. Cell Biology International 32, 549–553. 18158257 10.1016/j.cellbi.2007.11.007

[CIT0024] Pei W Du F Zhang Y He T Ren H 2012 Control of the actin cytoskeleton in root hair development. Plant Science 187, 10–18. 22404828 10.1016/j.plantsci.2012.01.008

[CIT0025] Peng J Ilarslan H Wurtele ES Bassham DC 2011 AtRabD2b and AtRabD2c have overlapping functions in pollen development and pollen tube growth. BMC Plant Biology 11, 25. 21269510 10.1186/1471-2229-11-25 PMC3040128

[CIT0026] Pereira-Leal JB Seabra MC 2001 Evolution of the Rab family of small GTP-binding proteins. Journal of Molecular Biology 313, 889–901. 11697911 10.1006/jmbi.2001.5072

[CIT0027] Pfeffer SR 2005 Structural clues to Rab GTPase functional diversity. Journal of Biological Chemistry 280, 15485–15488. 15746102 10.1074/jbc.R500003200

[CIT0028] Pfeffer SR 2013 Rab GTPase regulation of membrane identity. Current Opinion in Cell Biology 25, 414–419. 23639309 10.1016/j.ceb.2013.04.002 PMC3729790

[CIT0029] Preuss ML Schmitz AJ Thole JM Bonner HK Otegui MS Nielsen E 2006 A role for the RabA4b effector protein PI-4Kbeta1 in polarized expansion of root hair cells in Arabidopsis thaliana. Journal of Cell Biology 172, 991–998. 16567499 10.1083/jcb.200508116 PMC2063757

[CIT0030] Preuss ML Serna J Falbel TG Bednarek SY Nielsen E 2004 The Arabidopsis Rab GTPase RabA4b localizes to the tips of growing root hair cells. The Plant Cell 16, 1589–1603. 15155878 10.1105/tpc.021634 PMC490048

[CIT0031] Pylypenko O Rak A Reents R 2003 Structure of Rab escort protein-1 in complex with Rab geranylgeranyltransferase. Molecular Cell 11, 483–494. 12620235 10.1016/s1097-2765(03)00044-3

[CIT0032] Quilichini TD Douglas CJ Samuels AL 2014 New views of tapetum ultrastructure and pollen exine development in *Arabidopsis thaliana* . Annals of Botany (in press). 10.1093/aob/mcu042 PMC4195548 24723448

[CIT0033] Rak A Pylypenko O Niculae A Pyatkov K Goody RS Alexandrov K 2004 Structure of the Rab7:REP-1 complex: insights into the mechanism of Rab prenylation and choroideremia disease. Cell 117, 749–760. 15186776 10.1016/j.cell.2004.05.017

[CIT0034] Rasteiro R Pereira-Leal JB 2007 Multiple domain insertions and losses in the evolution of the Rab prenylation complex. BMC Evolutionary Biology 7, 140. 17705859 10.1186/1471-2148-7-140 PMC1994686

[CIT0035] Rossi G Yu JA Newman AP Ferro-Novick S 1991 Dependence of Ypt1 and Sec4 membrane attachment on Bet2. Nature 351, 158–161. 1903184 10.1038/351158a0

[CIT0036] Rutherford S Moore I 2002 The Arabidopsis Rab GTPase family: another enigma variation. Current Opinion in Plant Biology 5, 518–528. 12393015 10.1016/s1369-5266(02)00307-2

[CIT0037] Samaj J Muller J Beck M Bohm N Menzel D 2006 Vesicular trafficking, cytoskeleton and signalling in root hairs and pollen tubes. Trends in Plant Science 11, 594–600. 17092761 10.1016/j.tplants.2006.10.002

[CIT0038] Shirakawa M Ueda H Shimada T Koumoto Y Shimada TL Kondo M Takahashi T Okuyama Y Nishimura M Hara-Nishimura I 2010 Arabidopsis Qa-SNARE SYP2 proteins localized to different subcellular regions function redundantly in vacuolar protein sorting and plant development. The Plant Journal 64, 924–935. 21143674 10.1111/j.1365-313X.2010.04394.x

[CIT0039] Stenzel I Ischebeck T Konig S Holubowska A Sporysz M Hause B Heilmann I 2008 The type B phosphatidylinositol-4-phosphate 5-kinase 3 is essential for root hair formation in Arabidopsis thaliana. The Plant Cell 20, 124–141. 18178770 10.1105/tpc.107.052852 PMC2254927

[CIT0040] Synek L Schlager N Elias M Quentin M Hauser MT Zarsky V 2006 AtEXO70A1, a member of a family of putative exocyst subunits specifically expanded in land plants, is important for polar growth and plant development. The Plant Journal 48, 54–72. 16942608 10.1111/j.1365-313X.2006.02854.x PMC2865999

[CIT0041] Szumlanski AL Nielsen E 2009 The Rab GTPase RabA4d regulates pollen tube tip growth in Arabidopsis thaliana. The Plant Cell 21, 526–544. 19208902 10.1105/tpc.108.060277 PMC2660625

[CIT0042] Thole JM Nielsen E 2008 Phosphoinositides in plants: novel functions in membrane trafficking. Current Opinion in Plant Biology 11, 620–631. 19028349 10.1016/j.pbi.2008.10.010

[CIT0043] Thole JM Perroud PF Quatrano RS Running MP 2014 Prenylation is required for polar cell elongation, cell adhesion, and differentiation in Physcomitrella patens. The Plant Journal 78, 441–451. 24634995 10.1111/tpj.12484

[CIT0044] Thole JM Vermeer JE Zhang Y Gadella TW Jr Nielsen E 2008 Root hair defective4 encodes a phosphatidylinositol-4-phosphate phosphatase required for proper root hair development in Arabidopsis thaliana. The Plant Cell 20, 381–395. 18281508 10.1105/tpc.107.054304 PMC2276440

[CIT0045] Toufighi K Brady SM Austin R Ly E Provart NJ 2005 The Botany Array Resource: e-Northerns, Expression Angling, and promoter analyses. The Plant Journal 43, 153–163. 15960624 10.1111/j.1365-313X.2005.02437.x

[CIT0046] Ueda T Yamaguchi M Uchimiya H Nakano A 2001 Ara6, a plant-unique novel type Rab GTPase, functions in the endocytic pathway of *Arabidopsis thaliana* . EMBO Journal 20, 4730–4741. 11532937 10.1093/emboj/20.17.4730 PMC125591

[CIT0047] Uemura T Kim H Saito C Ebine K Ueda T Schulze-Lefert P Nakano A 2012 Qa-SNAREs localized to the trans-Golgi network regulate multiple transport pathways and extracellular disease resistance in plants. Proceedings of the National Academy of Sciences, USA 109, 1784–1789. 10.1073/pnas.1115146109 PMC3277133 22307646

[CIT0048] Voigt B Timmers AC Samaj J 2005 Actin-based motility of endosomes is linked to the polar tip growth of root hairs. European Journal of Cell Biology 84, 609–621. 16032929 10.1016/j.ejcb.2004.12.029

[CIT0049] Wu YW Goody RS Abagyan R Alexandrov K 2009 Structure of the disordered C terminus of Rab7 GTPase induced by binding to the Rab geranylgeranyl transferase catalytic complex reveals the mechanism of Rab prenylation. Journal of Biological Chemistry 284, 13185–13192. 19240028 10.1074/jbc.M900579200 PMC2676050

[CIT0050] Zhang Y McCormick S 2010 The regulation of vesicle trafficking by small GTPases and phospholipids during pollen tube growth. Sexual Plant Reproduction 23, 87–93. 20490965 10.1007/s00497-009-0118-z

