# Supplementary material for: Rab geranylgeranyl transferase β subunit is essential for male fertility and tip growth in Arabidopsis
Source: J Exp Bot. 2014 Oct 14;66(1):213–24. doi: 10.1093/jxb/eru412 (PMC4265159; doi:10.1093/jxb/eru412)
Supplement: Supplementary Data [file supp_66_1_213__index.html]

Rab geranylgeranyl transferase β subunit is essential for male fertility and tip growth in Arabidopsis — Rab geranylgeranyl transferase β subunit is essential for male fertility and tip growth in Arabidopsis — Supplementary Data 

# Rab geranylgeranyl transferase β subunit is essential for male fertility and tip growth in *Arabidopsis*

## Supplementary Data

Data files

**Files in this Data Supplement:**

- Supplementary Data - Supplementary Data
