# Supplementary material for: Rab geranylgeranyl transferase β subunit is essential for male fertility and tip growth in Arabidopsis
Source: J Exp Bot. 2014 Oct 14;66(1):213–24. doi: 10.1093/jxb/eru412 (PMC4265159; doi:10.1093/jxb/eru412)
Supplement: Supplementary Data [file supp_eru412_jexbot132753_file001.pdf]

Fig. S1

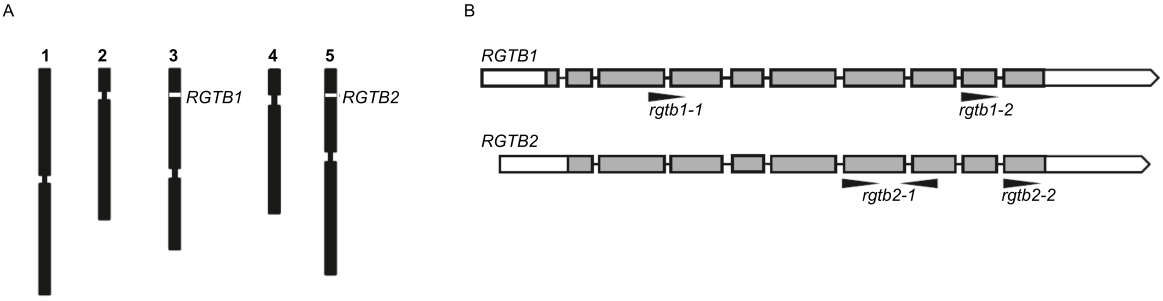

C

```
RGTB1   1 ATGAGCTCTACGTCTTCCTCCCAGATGGTTCAGCTCGTAGCTGACAAGCATGTACGTTACATTCT------GATGGCAGAAAA  77
RGTB2   1 ATGG-----------------CAGAC----AAGCTCGTAGCTGGCAAGCATCTTCGTTACATTCTGAATTTGATGGCAGAAAA  62

RGTB1  78 GAAGAAAGAGAGTTTTGAGTCTGTGGTGATGGATCATCTAAGAATGAATGGTGCATACTGGGGACTCACCACTCTCGACTTAC 160
RGTB2  63 GAAAAAAGAGAGTTTTGAGTCTGTGGTTATGGATCATCTGAGAATGAATGGTGCATATTGGGGACTAACCACACTGGCTTTAC 145

RGTB1 161 TTGACAAGCTCGGCTGTGTTTCTGAAGAGGAAGTCATTTCATGGCTCATGACTTGCCAGCATGAATCTGGTGGCTTTGCCGGT 243
RGTB2 146 TCGACAAGCTTGGTTCTGTTTCCGAAGATGAAGTTGTGTCATGGGTCATGACCTGCCAGCATGAGTCTGGTGGCTTTGCCGGT 228

RGTB1 244 AACACTGGACACGATCCACATATCCTATATACACTCAGTGCTGTACAAATCTTGGCCCTATTTGACAAAATCAACATTCTTGA 326
RGTB2 229 AATACTGGCCATGATCCGCATGTTTTGTATACACTCAGTGCTGTACAAATCTTGGCCCTCTTCGACAAACTCAACATTCTTGA 311

RGTB1 327 CATTGGGAAAGTGTCAAGTTATGTTGCTAAGTTGCAGAATGAAGATGGATCCTTCTCAGGAGATATGTGGGGAGAAATAGATA 409
RGTB2 312 TGTTGAAAAAGTTTCAAATTATATTGCTGGGTTACAGAATGAAGATGGATCCTTCTCAGGAGATATATGGGGCGAAGTAGATA 394

RGTB1 410 CGAGGTTTTCGTACATAGCTATTTGCTGTCTCTCAATATTGAAATGTCTTGACAAAATCAATGTGGAGAAGGCCGTTAAGTAC 492
RGTB2 395 CAAGGTTTTCTTACATAGCTATATGCTGCCTCTCAATATTAAAATGTCTTGATAAAATCAATGTGAAGAAGGCTGTTGATTAC 477

RGTB1 493 ATTGTGAGCTGCAAAAACCTGGATGGTGGTTTTGGGTGCACACCTGGAGCAGAGTCCCATGCAGGACAAATTTTCTGCTGCGT 575
RGTB2 478 ATTGTGAGCTGCAAAAACCTGGATGGTGGTTTTGGGTGCTCACCTGGAGCGGAGTCTCATGCAGGACAGATTTTCTGTTGTGT 560

RGTB1 576 TGGTGCTCTTGCTATCACTGGGAGTCTCCATCATGTTGATAAGGACTCACTTGGTTGGTGGTTATGTGAAAGACAA--TTA-A 655
RGTB2 561 GGGTGCTCTTGCTATCACCGGGAATCTCCATCGTGTTGATAAGGACTTACTTGGATGGTGGTTATGTGAAAGACAAGACTACG 643

RGTB1 656 AGGCTGGGGGCTTAAATGGACGTCCTGAGAAACTCGCTGATGTTTGCTATTCTTGGTGGGTCCTATCGAGCCTAATCATGATA 738
RGTB2 644 AGTCTGGAGGTTTAAATGGACGACCTGAGAAACTCCCTGATGTTTGCTATTCTTGGTGGGTCTTATCCAGCTTAATCATGATC 726

RGTB1 739 GATAGAGTTCACTGGATCGACAAAGCAAAGCTTGTCAAGTTCATTCTGGACTGTCAGGATTTGGACAACGGAGGCATCTCAGA 821
RGTB2 727 GATAGAGTTCACTGGATCGAGAAAGCAAAGCTTGTCAAGTTTATCTTGGACTCTCAGGACATGGATAACGGAGGCATCTCAGA 809

RGTB1 822 CCGACCCGAGGATGCTGTTGATATCTTCCACACCTACTTTGGAGTTGCGGGGCTTTCCCTCCTTGAGTATCCTGGAGTCAAAG 904
RGTB2 810 TAGACCCAGCTATACTGTTGATATCTTCCACACCTACTTTGGAGTTGCGGGGCTCTCTCTTCTCGAGTATCCTGGAGTCAAAA 892

RGTB1 905 TAATTGATCCAGCCTATGCTTTGCCTGTTGATGTCGTCAACCGGATTATATTCACCAAATGA 966
RGTB2 893 CAATTGATCCTGCCTACGCTTTGCCTGTCCATGTCATCAACCGGATTCTCTTCACCAAATGA 954
```

D

```
RGTB1   1 MSSTSSSQMVQLVADKHVRYIL--MAEK-KESFESVVMDHLRMNGAYWGLTTLDLLDKLGCVSEEEVISWLMTCQHESGGF  78
RGTB2   1 MAD-------KLVAGKHLRYILNLMAEKKKESFESVVMDHLRMNGAYWGLTTLALLDKLGSVSEDEVVSWVMTCQHESGGF  74

RGTB1  79 AGNTGHDPHILYTLSAVQILALFDKINILDIGKVSSYVAKLQNEDGSFSGDMWGEIDTRFSYIAICCLSILKCLDKINVEK 159
RGTB2  75 AGNTGHDPHVLYTLSAVQILALFDKLNILDVEKVSNYIAGLQNEDGSFSGDIWGEVDTRFSYIAICCLSILKCLDKINVKK 155

RGTB1 160 AVKYIVSCKNLDGGFGCTPGAESHAGQIFCCVGALAITGSLHHVDKDSLGWWLCERQ-LKAGGLNGRPEKLADVCYSWWVL 239
RGTB2 156 AVDYIVSCKNLDGGFGCSPGAESHAGQIFCCVGALAITGNLHRVDKDLLGWWLCERQDYESGGLNGRPEKLPDVCYSWWVL 236

RGTB1 240 SSLIMIDRVHWIDKAKLVKFILDCQDLDNGGISDRPEDAVDIFHTYFGVAGLSLLEYPGVKVIDPAYALPVDVVNRIIFTK 320
RGTB2 237 SSLIMIDRVHWIEKAKLVKFILDSQDMDNGGISDRPSYTVDIFHTYFGVAGLSLLEYPGVKTIDPAYALPVHVINRILFTK 317
```

Comparison of AtRGTB subunits. A) chromosomal location of AtRGTB genes, B) gene structure and location of inserts in corresponding SALK lines, C) comparison of nucleotide and D) aminoacid sequences of AtRGTB. Homology regions are shaded.

Amino acid and nucleotide sequence analysis and insert positioning

AtRGTB1 and AtRGTB2 nucleotide sequences, NM_121259 and NM_180236, respectively, were derived from the EMBL server. Corresponding amino acid sequences, NP_568259 and NP_850567, respectively, were derived from the PDB server. Alignments were performed with the T-coffee (http://www.ebi.ac.uk/Tools/msa/tcoffee) and visualized with Jalview. DNA obtained from genotyping PCR reaction of each mutant line was sequenced from the Lbb1.3 primer (http://oligo.ibb.waw.pl). Results of the sequencing were aligned with genomic sequence of the AtRGTB1 and AtRGTB2 genes with the EMBOSS program (http://emboss.bioinformatics.nl).

Fig. S2

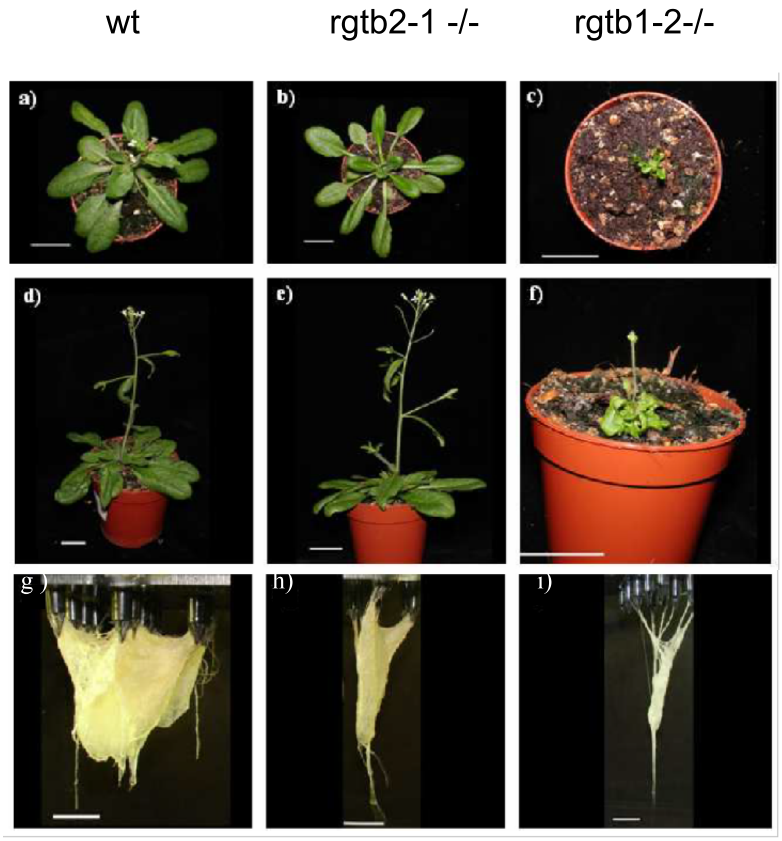

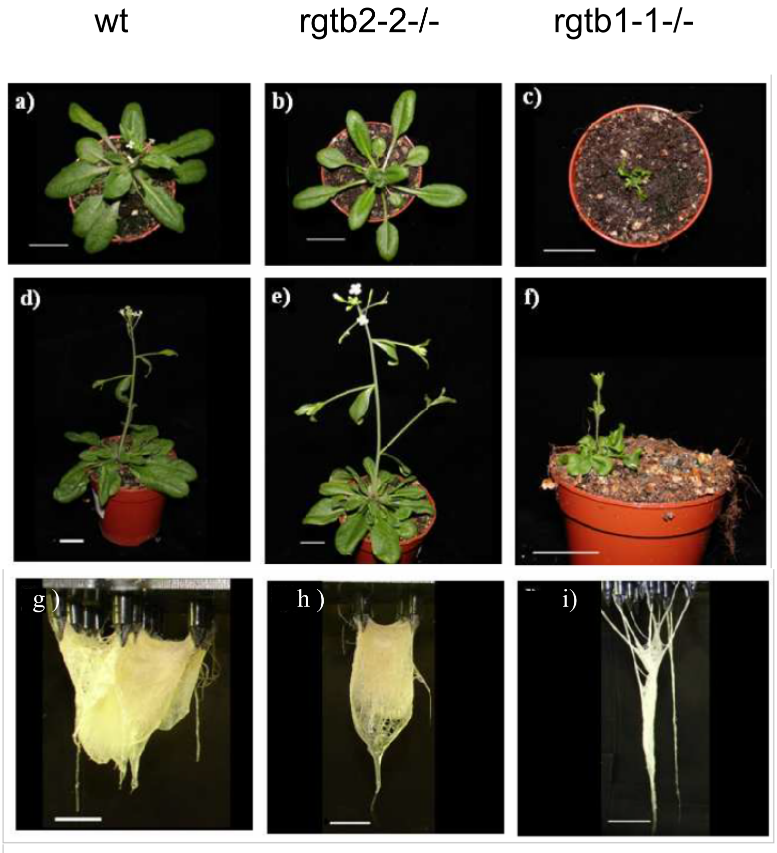

Fig. S2 Morphology of rgtb homozygous mutants. a-c) 4-week old rosette,
d-f) 5-week old flowering stem,
g-i) 5-week old root system grown in hydroponics.
